# Tumor stage-dependent expression of autophagy proteins in adrenocortical carcinoma

**DOI:** 10.3389/fendo.2026.1726834

**Published:** 2026-05-18

**Authors:** Sofia B. Oliveira, Diana Sousa, Madalena Costa, Elisabete Rios, Tiago Nunes da Silva, Valeriano Leite, Sofia S. Pereira, Duarte Pignatelli

**Affiliations:** 1 Unit for Multidisciplinary Research in Biomedicine (UMIB), School of Medicine and Biomedical Sciences (ICBAS), University of Porto, Porto, Portugal; 2 Laboratory for Integrative and Translational Research in Population Health (ITR), Porto, Portugal; 3 Institute for Research and Innovation in Health - Institute of Molecular Pathology and Immunology of the University of Porto, Porto, Portugal; 4 Department of Endocrinology, Unidade Local de Saúde de São João, Porto, Portugal; 5 Faculdade de Medicina Dentária, Universidade Católica Portuguesa (UCP), Viseu, Portugal; 6 Center for Interdisciplinary Research in Health (CIIS), Viseu, Portugal; 7 Department of Pathology, Unidade Local de Saúde de São João, Porto, Portugal; 8 Department of Medicine, Faculty of Medicine, University of Porto, Porto, Portugal; 9 Department of Endocrinology, Instituto Português de Oncologia de Lisboa Francisco Gentil, Lisboa, Portugal

**Keywords:** adrenocortical carcinoma, autophagy, immunohistochemistry, LC3, molecular pathophysiology, p62/SQSTM1

## Abstract

**Introduction:**

Adrenocortical carcinoma (ACC) is a rare endocrine malignancy with poor yet heterogeneous prognosis, mostly due to its complex and incompletely understood molecular background. Autophagy is a multi-step catabolic process with a dual role in tumorigenesis, acting either as a tumor suppressor or promoter in a context-dependent manner. Therefore, our aim was to investigate the expression of key proteins involved in different autophagy steps in both adrenocortical adenomas (ACA) and ACC, to further understand the role of autophagy in this type of tumors, particularly its involvement in the pathophysiology of ACC.

**Methods:**

Autophagy status was evaluated in ACA (n=20) and ACC (n=29) by assessing the expression of autophagy-related protein 5 (ATG5), microtubule associated protein 1 light chain 3 beta (LC3B), and sequestosome 1 (p62/SQSTM1), using immunohistochemistry. Additionally, *in vitro* experiments, including migration and invasion assays, were conducted in JIL-2266 and H295R cell lines to investigate the impact of autophagic flux inhibition in ACC cell behavior.

**Results:**

LC3B punctate staining was present in 89% of ACC and 25% of ACA, with significantly higher LC3B expression in ACC compared to ACA (14.81 ± 2.26% *vs* 2.05 ± 1.00%, *p* < 0.0001). ACC with ENSAT stage of 1–2 exhibited significantly higher LC3B expression compared to ACC with ENSAT 3-4 (19.17 ± 1.05% *vs* 12.62 ± 4.00%, *p* = 0.02). Similarly, non-metastatic ACC showed a significantly higher percentage of LC3B positive cells than metastasized ACC (18.77 ± 3.52% *vs* 5.83 ± 1.70%, *p* = 0.004). *In vitro*, inhibition of autophagy significantly reduced cell migration and invasion in ACC cells. Higher cytoplasmic p62/SQSTM1 levels were found in ACC with advanced disease (ENSAT 3–4 *vs* ENSAT 1-2: 72.07 ± 3.61% *vs* 51.33 ± 9.24%, *p* = 0.02). No significant differences were observed for ATG5.

**Discussion:**

Our findings indicate a tumor stage-dependent role of autophagy in ACC and show that autophagy may play a role in ACC molecular pathophysiology. A punctate LC3B expression accumulation, associated with less aggressive malignant features, namely absence of metastasis, appears to result from a blockade at the late stages of autophagy. Whereas active autophagy may be associated with a more aggressive cellular phenotype in ACC, particularly by promoting migration and invasion.

## 1 Introduction

Adrenocortical tumors (ACT) are common neoplasms originating from the adrenal cortex, affecting approximately 3 to 10% of human population (1). Most ACT are benign and non-functioning and are, therefore, incidentally discovered during imaging performed for unrelated non-adrenal conditions (2). In contrast, adrenocortical carcinoma (ACC) is a rare malignancy usually associated with a poor, yet heterogeneous prognosis, with five-year overall survival rate ranging from 13% to 80% (3). The classic prognostic factors, including tumor stage assessed by the European Network for the Study of Adrenal tumors (ENSAT) staging system, resection status, and Ki-67 proliferation index, are not sufficient to overcome the variability of ACC progression and patients’ survival (4). Moreover, effective treatment options are limited. Surgical resection remains the only potentially curative approach, but it is frequently followed by local recurrence or distant metastasis (5, 6). Mitotane, the only approved pharmacological drug for ACC, has limited efficacy (7, 8). These clinical limitations largely reflect an incomplete understanding of ACC pathophysiology, reinforcing the need for a deeper insight into the molecular mechanisms underlying malignancy in ACT.

Autophagy is a highly dynamic, multi-step process that culminates in the lysosomal degradation and/or recycling of cellular components, such as proteins and organelles, thereby fulfilling metabolic requirements and maintaining cellular homeostasis (9). As a complex multi-step mechanism, it is tightly regulated by a network of autophagy-related proteins (ATG), among which ATG5, microtubule-associated protein 1 light chain 3 (LC3), and sequestosome 1 (p62/SQSTM1) play distinct but coordinated roles (10–12). Autophagosomes, a double-membrane structure that encloses the cellular components for degradation and/or recycling, represent the functional and structural hallmark of autophagy (13). ATG5 and LC3 are key players of the autophagosome formation at different steps, whereas p62/SQSTM1 functions as an autophagy receptor that links ubiquitinated proteins to LC3 promoting their degradation (14). The autophagosome is then fused with a lysosome, originating an autophagolysosome, where cargo is degraded by lysosomal hydrolases under acidic pH (15).

Alterations in autophagy status are frequently observed in various diseases, particularly in cancer (16). Autophagy has emerged as a crucial yet paradoxical mechanism in tumorigenesis and tumor progression, acting either as a tumor suppressor or promoter, depending on the context (17, 18). In the case of ACT, this dual role of autophagy remains poorly understood. To date, most studies have focused on the role of autophagy in the mechanism of action of pharmacological treatments in ACC (19). However, there is a significant lack of understanding regarding the expression status of autophagy-related proteins in both adrenocortical adenoma (ACA) and ACC. To our knowledge, only two studies have evaluated the expression of autophagy-related proteins in ACC and/or ACA tissue samples, and their findings have been contradictory (20, 21).

Therefore, we aimed to evaluate the expression of key players involved in different autophagy steps- ATG5, LC3B and p62/SQSTM1- in both ACA and ACC tissues, to further understand the role of autophagy in this type of tumor, particularly its involvement in the pathophysiology of ACC. In addition, to provide functional insights into the biological relevance of these findings, we further investigated the effect of autophagy blocking in migration and invasion of ACC cell lines.

## 2 Materials and methods

### 2.1 Expression of autophagy-related proteins in ACT tissues

#### 2.1.1 Case selection

Adrenal tissue samples were obtained during adrenalectomy from patients with ACT (N = 49), comprising malignant tumors (ACC, n=29) and benign tumors (ACA, n=20). A summary of patients and tumors characteristics is presented in **Table 1**. Among ACC patients, the median follow-up time was 41 months, and 71% of the patients had at least 24 months of follow-up. The 1- and 3-year OS rates were 86% and 76%, respectively. A summary of patients and tumors characteristics is presented in **Table 1**. This study was approved by the Ethics Committee of Unidade Local de Saúde de São João (Ethical approval code: CE 62-14), Instituto Português de Oncologia do Porto Francisco Gentil (Ethical approval code: CE 14/2020), and Instituto Português de Oncologia de Lisboa Francisco Gentil (Ethical approval code: UIC/1534).

**Table 1:** Adrenocortical carcinoma and adenoma patient and tumor characteristics.

Patient/tumor characteristics	ACC	ACA
n	29	20
Age at surgery (years) (range)	55.00 ± 1.90 (34-76)	53.50 ± 3.71 (22-76)
Sex F:M (n)	14:15	13:5
Tumor size (cm) (range)	10.76 ± 1.18 (2.7-20)	3.60 ± 0.22 (2.4-5)
Weiss tumor (range)	3-8	0-1
ENSAT stage^a^		
I – II	30%	NA
III – IV	70%	NA
Functionality^b^		
NF	22%	60%
Aldosterone	5%	0%
Glucocorticoids	44%	40%
Androgens	17%	0%
Estrogen	6%	0%
Cortisol + Androgens	6%	0%

ACA, adrenocortical adenoma; ACC, adrenocortical carcinoma; ENSAT, European Network for the Study of Adrenal Tumors; N, Not applicable; NF, Non-function. ^a^Tumor stage at the time of diagnosis according to the ENSAT classification. ^b^Tumor functionality was established based on clinical evaluation of signs and symptoms of hormone excess, along with hormonal work-up. Data represents mean ± standard error of the mean values with ranges.

#### 2.1.2 Immunohistochemistry

The detection of key autophagy proteins- ATG5, LC3B, and p62/SQSTM1- in ACC and ACA tissues was performed by immunohistochemistry (IHC).

For that, 3 µm formalin-fixed paraffin-embedded tissue sections were mounted on adhesive microscope slides (J7800AMNZ, Epredia Superfrost Plus, Germany). Tissue sections were deparaffinized in xylol, hydrated in graded alcohols and underwent antigen retrieval, as described in **Table 2**. After that, sections were rinsed in washing buffer (**Table 2**), followed by a treatment with 3% hydrogen peroxide (1.07210, Merck, Darmstadt, Germany) in methanol for 30 minutes to block endogenous peroxidase activity. For LC3B and p62/SQSTM1, sections were incubated with goat-serum (1:5; S-1000, Vector Laboratories, Newark, California) in 10% bovine serum albumin (BSA) for 20 minutes, at room temperature (RT). After that, tissue sections were incubated overnight, at 4°C, with the respective primary antibody as described in **Table 2**. For ATG5, the detection of immune reaction was performed by incubation for 60 minutes at RT with the commercial Dako Real™ EnVision™ Detection System (K5007, Dako, Glostrup, Denmark). While the avidin-biotin complex was used for the detection of immune reactions for LC3B and p62/SQSTM1. For that, tissue sections were incubated with biotinylated secondary antibody goat anti-rabbit (1:200; BA-1000, Vector Laboratories, Newark, California) for 30 minutes at RT, followed by 30 minutes incubation at RT with ABC (1:100; PK-4000, Vector Laboratories, Newark, California). 3,3*’***-**diaminobenzidine (DAB) (K3468, Dako, Glostrup, Germany) was used as chromogen and hematoxylin (1.09249.2500, Sigma-Aldrich, Darmstadt, Germany) as nuclear counterstaining. Finally, tissue sections were dehydrated in graded alcohols and xylene before being mounted. As positive controls, placenta, kidney and stomach tissues were used for ATG5, LC3B and p62/SQSTM1, respectively. The omission of primary antibody was used as negative control.

**Table 2:** Summary table of positive control, antigen retrieval, washing solution, dilution and signaling amplification used for each antibody used.

IHC protocol parameters	ATG5 (MA5-35339, Invitrogen, Waltham, Massachusetts, USA)	LC3B (ab192890, Abcam, Cambridge, UK)	p62/SQSTM1 (ab109012, Abcam, Cambridge, UK)
Positive control	Placenta	Kidney	Stomach
Antigen retrieval	Pressure cooking boiling for 3 minutes in 0.01M citrate buffer pH 6.0 with 0.05% Tween 20	Microwave in 0.01M citrate buffer pH 6.0 with 0.05% Tween 20 for 15 minutes	Pressure cooking boiling for 3 minutes in 0.01M citrate buffer pH 6.0 with 0.05% Tween 20
Washing solution	PBS 0.05% Tween 20	PBS 0.05% Tween 20	PBS 0.05% Tween 20
Dilution	1:100	1:750	1:750
Signaling amplification	Chain polymer – conjugated technology	Avidin-Biotin Complex	Avidin-Biotin Complex

ATG5, autophagy-related protein 5; IHC, immunohistochemistry; LC3B, microtubule – associated protein light chain 3 beta; PBS, phosphate buffered-saline buffer; p62/SQSTM1, Sequestosome 1.

#### 2.1.3 Immunohistochemistry staining analysis

The immunostaining of ATG5, LC3B and p62/SQSTM1 was quantitatively assessed based on the percentage of positively stained cells within tumor hot spots. For each marker, slides were scanned using the image acquisition Olympus^®^ VS110™ (Olympus Corporation, Tokyo, Japan) virtual slide scanning system and captured using the image acquisition software VS-ASW (version 2.3 for Windows). Entire tumor sections were scanned using Olyvia software (Version 2.7, Olympus Corporation, Tokyo, Japan) to identify tumor hot spots for each autophagic protein. These regions were captured at 400x magnification, and positively stained cells were manually counted using ImageJ (National Institutes of Health, Bethesda, Maryland, USA), with a minimum of 1000 cells analyzed per ACC and ACA. The percentage of positive cells was calculated as the ratio of stained cells to the total number of cells analyzed. ATG5 positivity was identified by cytoplasmic staining. For LC3B, only cells showing a punctate staining pattern - previously reported to be directly associated with autophagy (22) - were considered positive. p62/SQSTM1 expression was detected in both the cytoplasm and nucleus of ACC and ACA cells. Accordingly, the two staining patterns were quantified separately, and the percentages of cells displaying nuclear and cytoplasmic p62/SQSTM1 positivity were calculated and reported independently. The interpretation and quantification of autophagic markers immunostaining were performed by one researcher and reviewed by two additional researchers and a pathologist.

The percentage of ATG5, LC3B, cytoplasmic p62/SQSTM1 stained cells was further categorized using a previously established immunostaining score system: score 0, negative or <1% positive cells; score 1, 1-10%; score 2, 11-50%; score 3, 51-100% positive cells (20). Nuclear p62/SQSTM1 staining was evaluated using a separate scoring system as follows: score 0 for nuclear staining in <10% of the cells and score 1 for ≥10% of the cells (13, 23).

### 2.2 Study of the effect of autophagy inhibition in cell migration and invasion of ACC cells

#### 2.2.1 Cell culture

The human adrenocortical carcinoma cell lines, JIL-2266 and H295R were used as *in vitro* models in this study. JIL-2266 was kindly provided by Laura-Sophie Landwehr (University of Würzburg, Germany" to "University Hospital of Würzburg, Germany), while the H295R cell line was obtained from CLS Cell Lines Service GmbH (Eppelheim, Germany).

JIL-2266 cells were grown in 3:1 (v/v) Dulbecco’s Modified Eagle Medium (DMEM) - high glucose (Gibco, Thermo Fisher Scientific, Waltham, MA, USA) and Nutrient Mixture F-12 (Invitrogen, Waltham, MA, USA), supplemented with 10% Fetal Bovine Serum (FBS; Gibco, Thermo Fisher Scientific, Waltham, MA, USA), insulin (Sigma-Aldrich, St. Louis, MO, USA), hydrocortisone (Sigma-Aldrich, St. Louis, MO, USA), cholera toxin (Sigma-Aldrich, St. Louis, MO, USA), adenine (Sigma-Aldrich, St Louis, MO, USA) epidermal growth factor (EGF; Invitrogen, Thermo Fisher Scientific, Waltham, MA, USA), and penicillin-streptomycin (Sigma-Aldrich, St. Louis, MO, USA). H295R cells were cultured in DMEM: F-12 (Sigma-Aldrich, St Louis, MO, USA) supplemented with 2.5% Nu-Serum (BD Bioscience, San Jose, CA), L-Glutamine (Sigma-Aldrich, St. Louis, MO, USA), Insulin-Transferrin-Selenium Premix (ITS; Corning, NY, USA) and penicillin-streptomycin (Gibco, Thermo Fisher Scientific, Waltham, MA, USA). Cell culture was maintained in a humidified incubator at 37 °C with 5% CO_2._

#### 2.2.2 Analysis of the effect of autophagy inhibition in cellular density and in expression of autophagic proteins

JIL-2266 (0.25 x 10^6^ cells/well) and H295R (0.65 x 10^6^ cells/well) cells were plated in six-well plates and allowed to adhere for 24 h. JIL-2266 cells were then treated for 48h with Bafilomycin A1 (BafA1; B1793, Sigma-Aldrich, St. Louis, MO, USA), a late-stage autophagic flux inhibitor, at two different concentrations (10 nM and 20 nM), previously described as effective concentrations for autophagy blockade (24–27). Appropriate controls were included: untreated cells (blank), cells treated with DMSO (vehicle) corresponding to the volume used for BafA1 (10 nM and 20 nM) condition, and a positive control for autophagy induction using EBSS (Sigma-Aldrich, St. Louis, MO, USA).

To avoid confounding effects of reduced cell number on migration and invasion results, we first established BafA1 concentrations (10 and 20 nM, 48 h) that effectively modulate autophagic flux without significantly affecting cell density in JIL-2266 and H295R cells. Accordingly, both autophagic flux and cellular density were evaluated prior to performing migration and invasion assays.

After cell treatment with BafA1 and the respective controls, cell density was assessed for each condition using Trypan-Blue Exclusion assay (T8154, Sigma-Aldrich, St Louis, MO, USA). For autophagic flux assessment, cells were lysed in RIPA buffer (1% NP-40, 0.5% Sodium deoxycholate, 0.1% SDS 10%, PBS) supplemented with a protease inhibitor cocktail (4693124001, Roche, Switzerland). Protein lysates were quantified using the Pierce BCA Protein Assay Kit (23225, Thermo Fisher Scientific, Rockford, USA) according to the manufacturer’s instructions. A total of 20 µg of protein per sample, pre-heated at 95 °C for 5 minutes, were loaded and separated by size on a 15% sodium dodecyl sulfate polyacrylamide gel electrophoresis (SDS-PAGE). Through a Trans-Blot Turbo Transfer System (1.3 A, up to 25 V, 7 min; Bio‐Rad, Hercules, California, USA), protein samples were transferred to nitrocellulose membranes (Bio‐Rad, Hercules, California, USA) followed by 1 h blocking at RT in 5% of dried milk, 0.05% Tween 20 in Tris-buffered saline solution (TBS). Then, the blocked membranes were incubated, separately, with the primary antibodies at 4 °C overnight: rabbit anti-LC3B (1:2500; ab192890, Abcam, Cambridge, UK) rabbit anti-p62/SQSTM1 (1:10 000; ab109012, Abcam, Cambridge, UK) and mouse anti-GAPDH (1:10 000; ab8245, Abcam, Cambridge, UK), used as loading control. On the next day, the membranes were washed with 0.05% Tween 20 in TBS at RT, and incubated with the correspondent peroxidase-conjugated secondary antibodies, goat anti-rabbit (1:10 000; ab6721, Abcam, Cambridge, UK) and goat anti-mouse (1:20 000, ab97040, Abcam, Cambridge, UK), for 1 h at RT. Finally, ECL (Bio‐Rad, Hercules, California, USA) was added to the membranes to visualize protein bands in a ChemiDoc MP Imaging System (Bio-Rad, Hercules, California, USA). The densities of each band were quantified using the Image Lab software (Bio‐Rad, Hercules, California, USA).

These experiments were independently repeated 3 times for each cell line.

#### 2.2.3 Wound healing assay

The wound healing assay was performed using the Ibidi Culture-Insert 2 Well (Ibidi GmbH, Gräfelfing, Germany). JIL-2266 (0.025 × 10^6^ cells) and H295R (0.042 × 10^6^ cells) were seeded into each well of the Culture-Insert. To ensure autophagic flux disruption prior to the migration assay, cells were pre-treated with 10 nM BafA1 for 4 h. Pre-treatment was initiated when cells reached at least 90% of confluence. The 4 h pre-treatment period was selected based on prior confirmation of impaired autophagic flux in both cell lines, as determined by LC3-II expression levels by Western blot (**Supplementary Figure 1**). After 4h, the Culture-Insert 2 Well was gently removed using sterile tweezers. The cell layer was washed with PBS to remove cell debris and nonattached cells, and BafA1 treatment (10 nM) or the corresponding vehicle (DMSO) was added for 48h. Images of the wound area were acquired using an inverted microscope (ECLIPSE Ts2, Nikon, The Nederlands) at different time-points: 0, 12, 24, 36, and 48 h. For each condition and timepoint, three randomly selected images were captured. Wound area was quantified using the “Wound_healing.ijm” macro in ImageJ as previously described (28). Wound area for each time-point is expressed as the area between cell monolayer borders normalized to the initial wound area (0 h), whereas wound closure rate was calculated as the ratio of the difference of wound area between consecutive 12 h intervals. These experiments were repeated 5 and 2 times for JIL-2266 and H295R cell lines, respectively.

#### 2.2.4 Invasion assay

To evaluate the impact of autophagy modulation on the invasion capacity of JIL-2266 and H295R cells, cell culture inserts with an 8.0 µm pore size (BioCoat^®^ Matrigel^®^ Invasion Chambers with 8.0 µm PET Membrane in 24-well Plates; Corning, NY, USA) were used according to the manufacturer’s instructions. Matrigel-coated inserts were pre-incubated with serum-free DMEM-F12 for 2 hours at 37 °C, before JIL-2266 (0.050 x 10^6^ cells/insert) and H295R (0.065 x 10^6^ cells/insert) were seeded in the upper chamber of the well and maintained through all assay in their respective media supplemented with 2% of FBS and 2.5% of Nu-Serum, respectively. After seeding, cells were left to adhere for 6 hours, followed by a 4 h pre-treatment with BafA1 (10 nM) and DMSO (vehicle) in the upper chamber, as described for the migration assay. After 4 h, medium supplemented with 20% FBS and 20% Nu-Serum was added to the lower chamber as a chemoattractant for JIL-2266 and H295R cells, respectively. As a negative control, medium containing 2% FBS or 2.5% Nu-Serum was added to the lower chamber (matching the upper chamber conditions) to eliminate the chemotactic gradient. After 48 h, the medium was collected, and the membranes of the inserts were fixed with 70% ethanol and the cells were stained with 0.2% crystal violet for 10 min, as previously described (29). The membranes were then mounted on slides using Entellan. Images of invaded cells were captured (Leica EC3 Microscope Digital Camera, Leica, Wetzlar, Germany) and invaded cells were quantified by manually counting cells in at least five randomly selected fields using ImageJ. All experiments were independently performed four times.

### 2.3 Statistical analysis

All ordinal data are represented as mean ± standard error of the mean (SEM). The normality for continuous variables was assessed using Shapiro-Wilk test. For comparisons between two groups, the unpaired *t*-test was used for normally distributed data [autophagy-related proteins positive cells (%) in ACC *vs* ACA: cytoplasmatic p62/SQSTM1; malignant parameters: ENSAT stage (cytoplasmatic and nuclear p62/SQSTM1), distant metastasis (cytoplasmatic and nuclear p62/SQSTM1), capsular invasion (nuclear p62/SQSTM1), venous invasion (LC3B, cytoplasmatic and nuclear p62/SQSTM1), sinusoidal invasion (LC3B, cytoplasmatic and nuclear p62/SQSTM1), Fuhrman Grade (LC3B, cytoplasmatic and nuclear p62/SQSTM1), tumor functionality (LC3B, cytoplasmatic and nuclear p62/SQSTM1), glucocorticoids secretion (LC3B, cytoplasmatic and nuclear p62/SQSTM1)], while the Mann-Whitney test was applied to non-normally distributed data [autophagy-related proteins positive cells (%) in ACC *vs* ACA: ATG5, LC3B, nuclear p62/SQSTM1; malignant parameters: ENSAT stage (ATG5, LC3B), distant metastasis (ATG5, LC3B), capsular invasion (ATG5, LC3B, cytoplasmatic p62/SQSTM1), venous invasion (ATG5), sinusoidal invasion (ATG5), Fuhrman Grade (ATG5), tumor functionality (ATG5), glucocorticoids secretion (ATG5); average wound closure rate in JIL-2266 (vehicle vs Bafilomycin A1 treatment); invaded cells (%) in JIL-2266 and H295R cells (vehicle *vs* Bafilomycin A1 treatment)]. Differences in categorical variables between groups were analyzed using the Chi-squared (χ^2^) (% of ACT cases (scores): ATG5, LC3B) or Fisher test (% of ACT cases (scores): cytoplasmatic and nuclear p62/SQSTM1). The correlation between continuous variables were evaluated using the Pearson (tumor size *vs* positive cells (%): LC3B, cytoplasmatic and nuclear p62/SQSTM1; autophagy-related proteins positive cells (%): cytoplasmatic *vs* nuclear p62/SQSTM1) or Spearman test (tumor size *vs* positive cells (%): ATG5; autophagy-related proteins positive cells (%): ATG5 *vs* LC3B, ATG5 *vs* cytoplasmatic p62/SQSTM1, ATG5 *vs* nuclear p62/SQSTM1, LC3B *vs* cytoplasmatic p62/SQSTM1, LC3B *vs* nuclear p62/SQSTM1, ATG5 *vs* nuclear p62/SQSTM1) as appropriate. Comparisons between time-points for wound closure area (in JIL-2266 cells) were performed using two-way ANOVA with Sidak’s *post hoc* test.

The area under the receiver operating characteristic (ROC) curve (AUC) was used to determine the diagnosis accuracy of the autophagy-related proteins markers (% of positive cells) in ACT. The Univariate Cox regression analysis was conducted to assess the association between the autophagy-related proteins expression with overall survival of ACC patients.

All statistical analyses were conducted using GraphPad Prism (Version 8.0, GraphPad Software, California, USA), except for the Cox regression analysis which was performed by using IBM SPSS (Version 29.0, New York, USA). A *p* < 0.05 was considered statistically significant.

## 3 Results

### 3.1 Expression of autophagy-related proteins in ACT tissues

#### 3.1.1 ATG5 expression is similar in ACC and ACA

**Figure 1:**
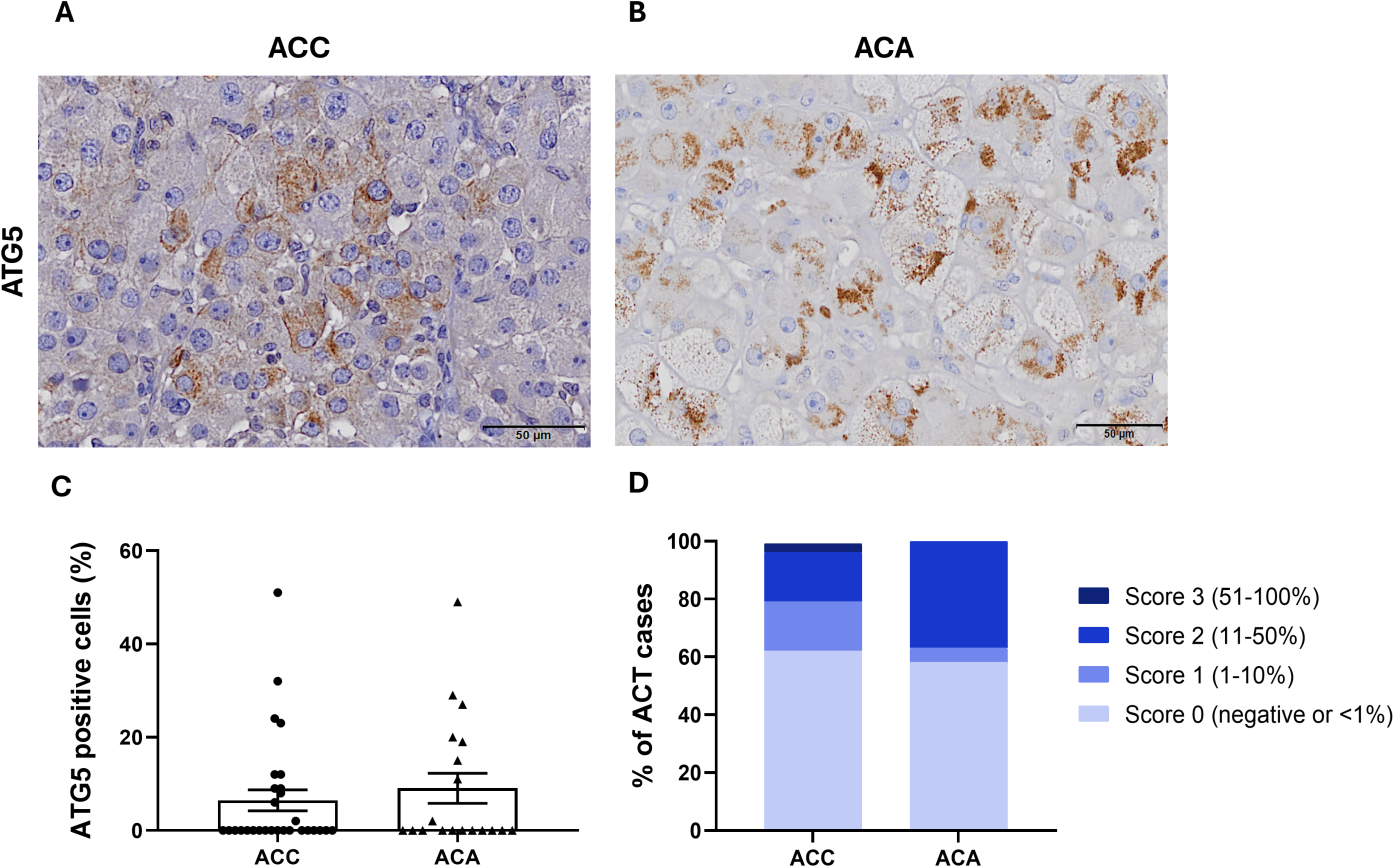
ATG5 immunostaining in **(A)** ACC, adrenocortical carcinoma and in **(B)** ACA, adrenocortical adenoma (400x). **(C)** Percentage of ATG5 positive cells in ACC and ACA (Mann-Whitney: non-significant). **(D)** Frequency of ACT, adrenocortical tumors with ATG5 expression across different categorization scores (0-3) (Chi-square test: non-significant).

ATG5 immunostaining was observed in the cytoplasm of both ACC and ACA cells (**Figures 1A, B**). Positive immunostaining for ATG5 was detected in 38% of ACC (11/29) and 42% of ACA (8/19) (**Figure 1C**).

The percentage of ATG5 stained cells was lower in ACC (6.48 ± 2.24%) compared to ACA (9.05 ± 3.21%), however no significant differences were observed between these two groups (**Figure 1C**). Only one ACC showed a percentage of ATG5 - positive cells above 50% (score 3) (**Figures 1C, D**).

#### 3.1.2 LC3B expression is higher in ACC than in ACA

**Figure 2:**
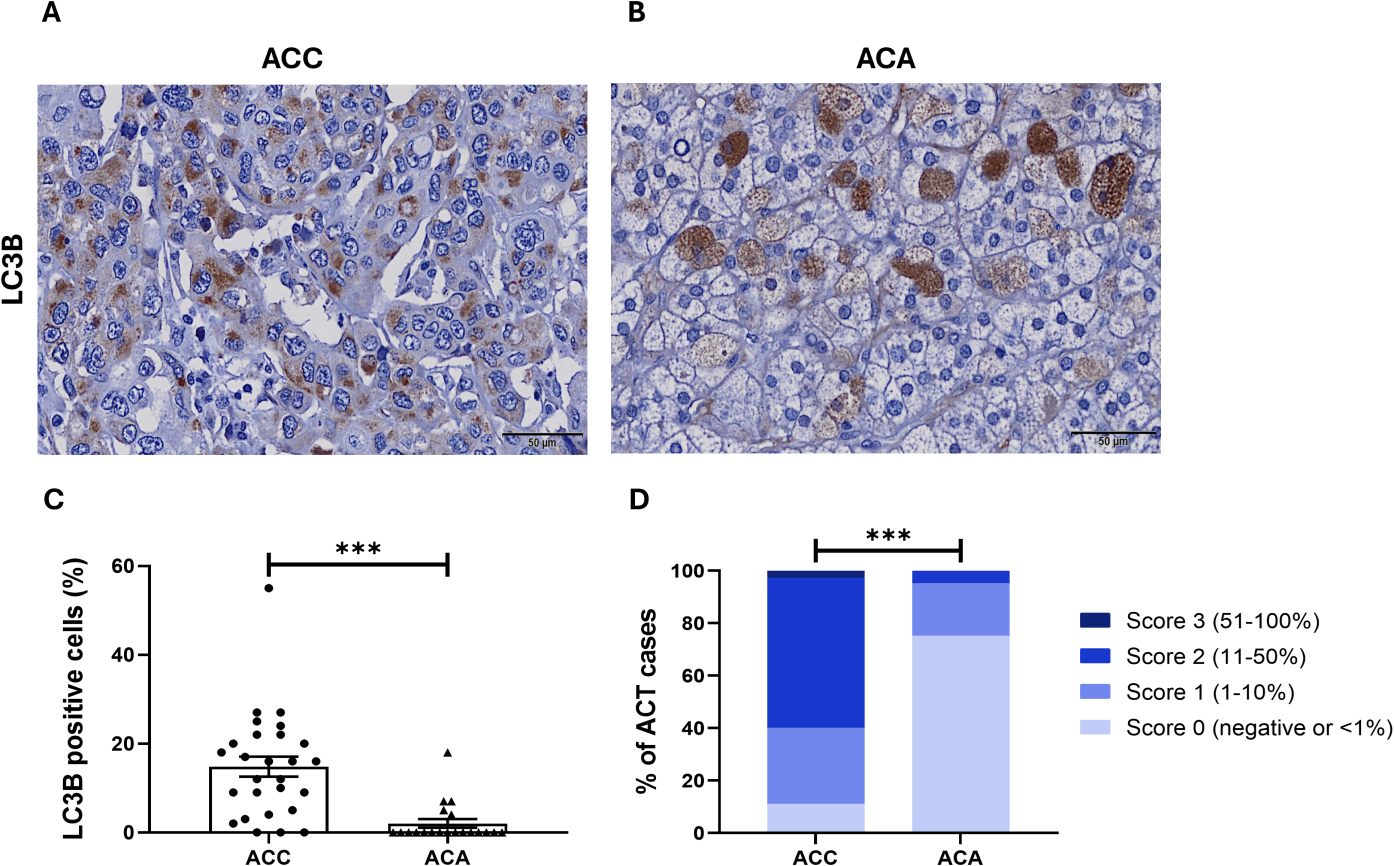
LC3B immunostaining in **(A)** ACC, adrenocortical carcinoma and in **(B)** ACA, adrenocortical adenoma (400x). **(C)** Percentage of LC3B positive cells in ACC and ACA (Mann Whitney: ***, p < 0.0001). **(D)** Frequency of ACT, adrenocortical tumors with LC3B expression across different categorization scores (0-3) (Chi-square test: ***, *p* < 0.0001).

A punctate LC3B staining pattern was present in 89% of ACC (24/27) and in 25% of ACA cases (5/20) (**Figures 2A-C**). In addition, the percentage of LC3B positive stained cells was significantly higher in ACC compared to ACA (ACC *vs* ACA: 14.81 ± 2.26% *vs* 2.05 ± 1.00%, *p* < 0.0001) (**Figure 2C**). Regarding score categorization, most of ACC had a score of 2 and 3, i.e., higher LC3B expression, whereas only one ACA had a score of 2 (**Figure 2D**).

#### 3.1.3 p62/SQSTM1 nuclear expression is lower in ACC than in ACA

**Figure 3:**
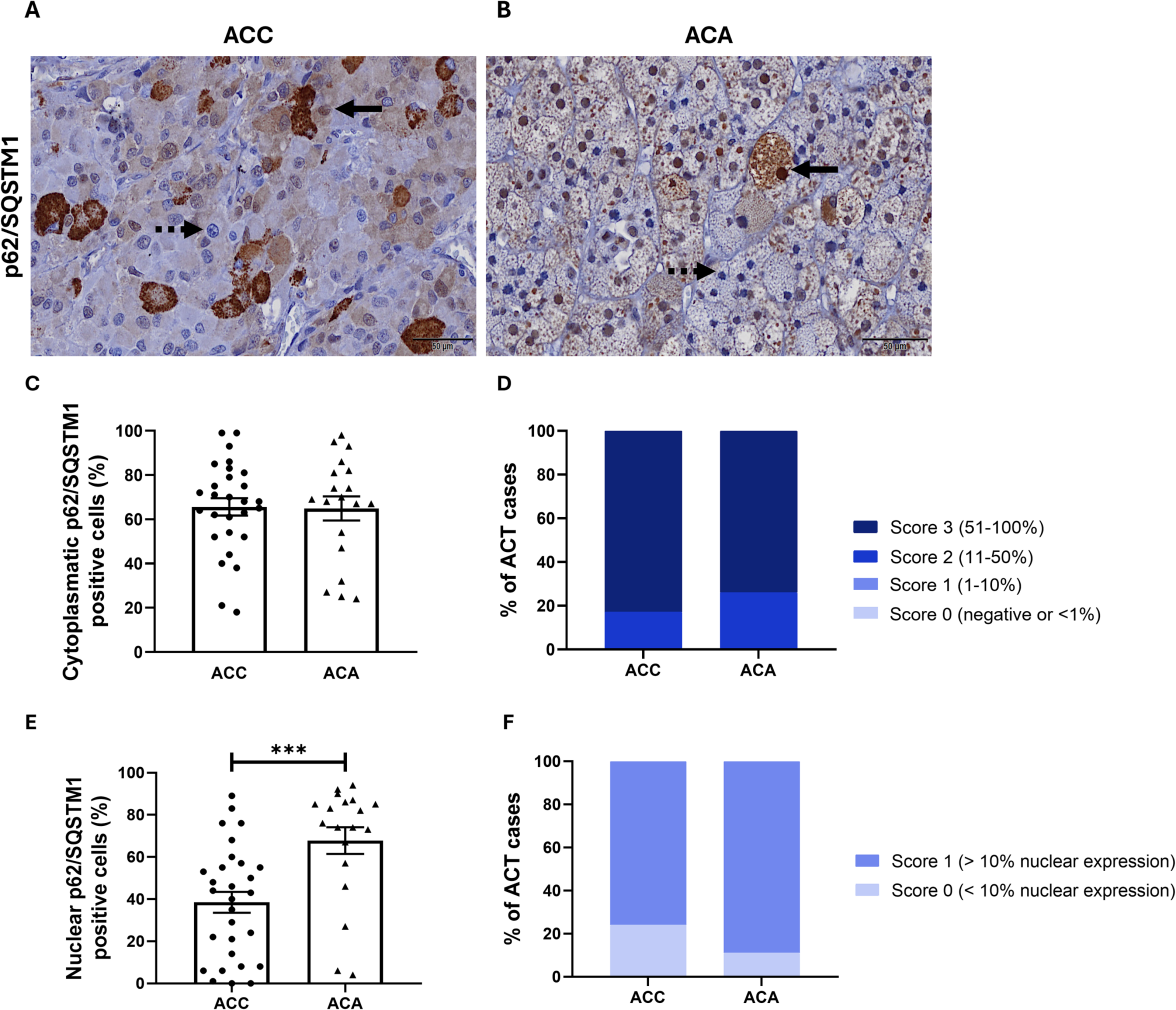
p62/SQSTM1 immunostaining in **(A)** ACC, adrenocortical carcinoma and **(B)** ACA, adenoma (400x), with solid and dashed arrows indicating examples of positive and negative nuclear p62/SQSTM1 staining, respectively. **(C)** Percentage of cytoplasmic p62/SQSTM1 positive cells in ACC and ACA (unpaired *t*-test: non-significant). **(D)** Frequency of ACT, adrenocortical tumors with cytoplasmic p62/SQSTM1 expression across different categorization scores (0-3) (Fisher test: non-significant). **(E)** Percentage of nuclear p62/SQSTM1 positive cells in ACC and ACA (Mann-Whitney: ***, p<0.001). **(F)** Frequency of ACT with nuclear p62/SQSTM1 expression categorized in scores (0-1) (Fisher test: non-significant).

p62/SQSTM1 immunostaining was observed both in the cytoplasm and in the nucleus of ACC and ACA cells (**Figures 3A, B**).

Cytoplasmic staining was present in all ACC and ACA samples. No significant differences were found in the percentage of p62/SQSTM1 positive cells between ACC and ACA (**Figure 3C**). In addition, cytoplasmic p62/SQSTM1 expression was consistently high across both ACC and ACA, indicating a homogenous staining pattern (score 2 and 3) (**Figure 3D**).

Nuclear p62/SQSTM1 staining was found in all ACA while only 2 ACC cases did not express p62/SQSTM1 in the nucleus (**Figure 3E**). However, ACC showed a significantly lower percentage of nuclear p62/SQSTM1 positive cells when compared to ACA (39.52 ± 4.96% *vs* 67.79 ± 6.35%, *p* < 0.001) (**Figures 3E, F**).

#### 3.1.4 ACC in advanced stages presents a lower LC3B expression and higher cytoplasmatic p62/SQSTM1 expression

Differences in the expression of ATG5, LC3B and p62/SQSTM1 (both cytoplasmic and nuclear) were evaluated regarding tumor parameters, including ENSAT tumor stage, presence of distant metastasis, capsular, venous and sinusoidal invasion, Fuhrman nuclear grade, tumor functionality, including a sub-analysis of autonomous glucocorticoids (GC) secretion (**Table 3**).

**Table 3:** Percentage of positive cells of each autophagic marker in adrenocortical carcinoma according to ENSAT score, metastasis, capsular, venous and sinusoidal invasion, Fuhrman nuclear grade, functionality and glucocorticoids secretion.

Tumor parameters		ATG5	LC3B	Cytoplasmatic p62/SQSTM1	Nuclear p62/SQSTM1
ENSAT stage	1-2	1.00 ± 1.00	19.17 ± 1.05	51.33 ± 9.24	19.83 ± 9.36
	3-4	10.93 ± 4.25	12.62 ± 4.00	72.07 ± 3.61	40.79 ± 6.75
	*p*	0.14	0.02*	0.02*	0.10
Distant metastasis	Absence	10.46 ± 4.63	18.77 ± 3.52	65.00 ± 5.98	36.00 ± 8.32
	Presence	3.29 ± 1.91	5.83 ± 1.70	67.43 ± 5.20	31.71 ± 6.68
	*p*	0.79	0.004**	0.79	0.73
Capsular invasion	Absence	2.00 ± 2.00	14.00 ± 6.00	80.00 ± 5.00	23.33 ± 12.45
	Presence	5.53 ± 2.31	14.94 ± 2.08	63.06 ± 4.53	34.71 ± 5.86
	*p*	0.59	0.93	0.11	0.56
Venous invasion	Absence	5.00 ± 3.92	14.50 ± 3.33	58.00 ± 10.93	16.33 ± 8.52
	Presence	2.60 ± 1.33	14.22 ± 3.11	62.67 ± 3.67	39.90 ± 6.87
	*p*	0.84	0.95	0.64	0.05
Sinusoidal invasion	Absence	2.50 ± 1.63	13.33 ± 3.98	63.17 ± 6.10	25.50 ± 11.93
	Presence	4.56 ± 2.72	14.63 ± 3.02	60.13 ± 7.80	31.56 ± 6.68
	*p*	0.79	0.79	0.78	0.64
Fuhrman grade	High	4.29 ± 3.40	16.71 ± 2.52	58.00 ± 8.83	22.86 ± 8.09
	Low	3.40 ± 2.09	13.20 ± 5.11	60.50 ± 8.27	47.20 ± 10.31
	*p*	0.86	0.51	0.86	0.09
Tumor functionality^a^	Yes	3.31 ± 1.91	11.31 ± 2.28	67.54 ± 4.32	32.54 ± 6.78
	No	15.75 ± 12.09	30.33 ± 13.38	78.50 ± 3.79	62.25 ± 12.02
	*p*	0.12	0.03*	0.20	0.04*
GC excess	Yes	3.89 ± 2.73	10.88 ± 2.81	63.33 ± 3.13	30.00 ± 7.24
	No	8.89 ± 5.47	21.63 ± 5.80	76.44 ± 5.41	47.00 ± 9.76
	*p*	0.29	0.12	0.07	0.18

ATG5, autophagy-related protein 5; ENSAT, European Network for the Study of Adrenal Tumors; GC, glucocorticoids; LC3B, microtubule – associated protein light chain 3 beta; p62/SQSTM1, Sequestosome 1. Statistically significant results in LC3B: ENSAT stage, Mann-Whitney: *, *p* < 0.05; Distant metastasis, Mann-Whitney: **, *p* < 0.01; Tumor functionality, unpaired *t*-test: *, *p* < 0.05; and in cytoplasmic p62/SQSTM1: ENSAT stage, unpaired *t*-test *, *p* < 0.05; p62/SQSTM1 nuclear: Tumor functionality, unpaired *t*-test *, *p* < 0.05. ^a^ Tumor functionality was established based on clinical evaluation of signs and symptoms of hormone excess, along with hormonal work-up.

ACC with ENSAT stage of 1–2 exhibited significantly higher LC3B expression when compared to ACC with ENSAT stage 3-4 (19.17 ± 1.05% *vs* 12.62 ± 4.00%, *p* = 0.02). Similarly, non-metastatic ACC showed a significantly higher percentage of LC3B positive cells than metastasized ACC (18.77 ± 3.52% *vs* 5.83 ± 1.70%, *p* = 0.004). In contrast, higher expression of cytoplasmic p62/SQSTM1 was found in ACC with advanced ENSAT stage (ENSAT 1–2 *vs* ENSAT 3-4: 51.33 ± 9.24% *vs* 72.07 ± 3.61%, *p* = 0.01) (**Table 3**).

Regarding tumor functionality, functioning ACC showed a significantly lower LC3 and nuclear p62/SQSTM1 expression compared to non-functioning ACC (LC3: 11.31 ± 2.28% *vs* 30.33 ± 13.38%, *p* = 0.03; nuclear p62/SQSTM1: 32.54 ± 6.78% *vs* 62.25 ± 12.02%, *p* = 0.04). However, no significant association was observed for GC secretion (**Table 3**).

No significant differences were observed between ATG5 expression and the analyzed features (**Table 3**).

In addition, no significant correlations were found between the autophagic proteins expression and tumor size or among autophagy-related proteins (r<0.3, *p*>0.05), except for a moderate positive correlation between ATG5 and p62/SQSTM1 nuclear expression in ACC (r=0.41, *p* = 0.02) (**Supplementary Table 1**).

#### 3.1.5 Autophagy-related proteins showed limited diagnostic value

LC3B and p62/SQSTM1 nuclear showed good and moderate discriminatory performance in distinguishing ACC from ACA, with AUC values of 0.88 and 0.79, respectively, however, no optimal cut-off value provided both high sensitivity and specificity (**Supplementary Figure 2**). ATG5 and p62/SQSTM1 cytoplasmatic failed to demonstrate diagnosis value for ACC, with AUC < 0.60 (**Supplementary Figure 2**).

#### 3.1.6 Autophagy-related proteins expression is not associated with patients’ overall survival

Using univariate Cox regression analysis, it was observed that the expression of ATG5, LC3B, and p62/SQSTM1 did not seem to influence overall survival of patients with ACC (**Table 4**).

**Table 4:** Univariate Cox regression analysis assessing the influence of the autophagy-related proteins expression on the overall survival of patients with adrenocortical carcinoma.

Autophagy-related proteins		Hazard ratio	95% CI	*p*
ATG5	% positive cells	1.04	0.98−1.10	0.18
	Score 0-1	1.82	0.33−9.97	0.49
	Score 2-3			
LC3B	% positive cells	1.05	0.97−1.14	0.22
	Score 0-1	0.66	0.13-3.25	0.61
	Score 2-3			
cytoplasmatic p62/SQSTM1	% positive cells	1.01	0.97−1.07	1.02
	Score 0-1	-^a^	-^a^	-^a^
	Score 2-3			
nuclear p62/SQSTM1	% positive cells	0.98	0.95−1.02	0.38
	Score 0	0.61	0.11−3.34	0.57
	Score 1			

ATG5, autophagy-related protein 5; LC3B, microtubule – associated protein light chain 3 beta; p62/SQSTM1, Sequestosome 1; ^a^All ACC included in the Cox regression analysis had a cytoplasmatic p62 expression score of 2-3.

### 3.2 Study of the effect of autophagy inhibition in cell migration and invasion of ACC cell lines

#### 3.2.1 Dose-dependent effects of BafA1 on autophagic flux and cell density in ACC cells

**Figure 4:**
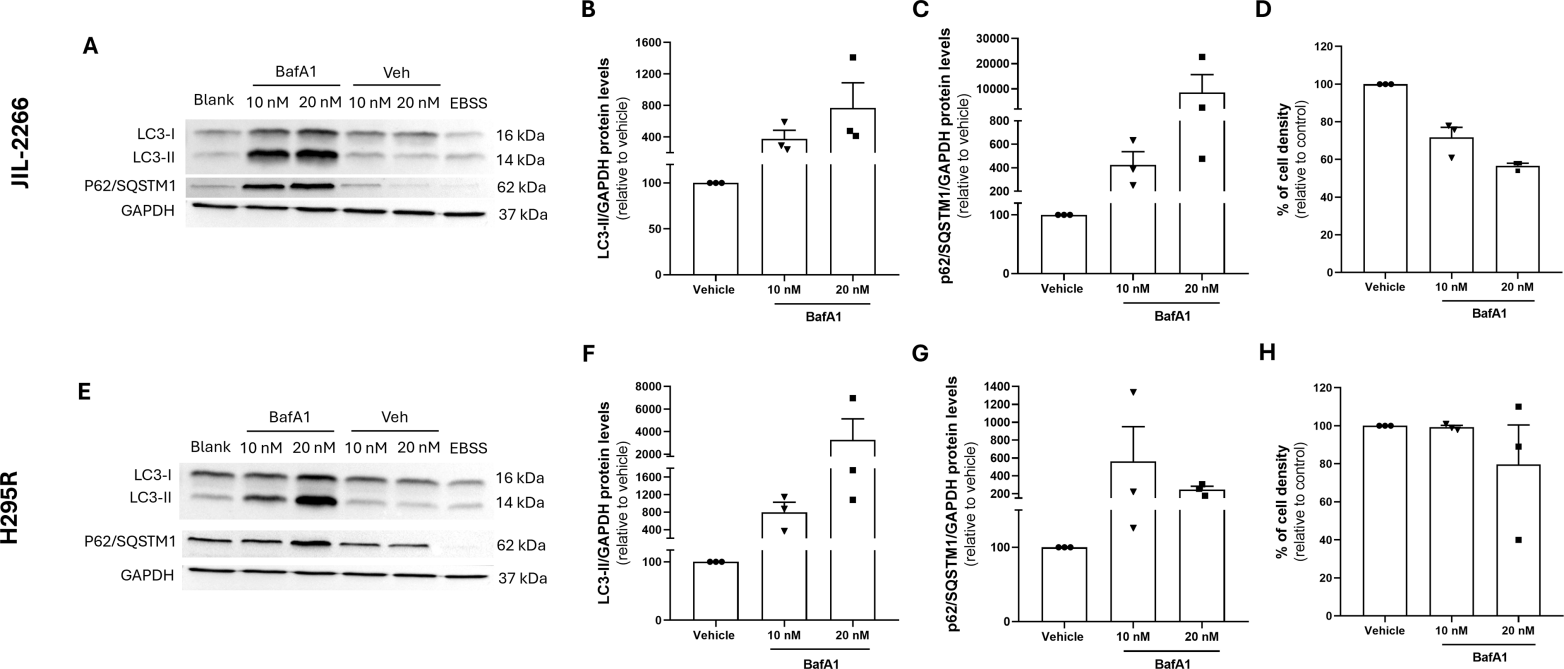
Effect of BafA1, Bafilomycin A1 on the autophagic flux and in cell density in JIL-2266 and H295R cell lines. Cells were treated for 48 h with medium (blank), BafA1 (10 or 20 nM), or with the corresponding DMSO (veh) concentrations, and with EBSS, an autophagy inducer. **(A)** Representative image of LC3-I, LC3-II, p62/SQSTM1 and GAPDH Western Blot in JIL-2266 cell line. **(B, C)** LC3-II and p62/SQSTM1 expression levels after BafA1 treatment (10 and 20 nM) normalized to GAPDH relative to vehicle, analyzed by Western Blot. **(D)** JIL-2266 cell density after 48h of BafA1 treatment assessed by Trypan-Blue Exclusion assay. **(E)** Representative image of LC3-I, LC3-II, p62/SQSTM1 and GAPDH Western Blot in H295R cell line. **(F, G)** LC3-II and p62/SQSTM1 expression levels after BafA1 treatment (10 and 20 nM) normalized to GAPDH relative to vehicle, analyzed by Western Blot. **(H)** H295R cell density after 48h BafA1 treatment (10 and 20 nM) assessed by Trypan-Blue Exclusion assay. Results are representative of 3 independent experiments for each cell line.

In both ACC cell lines, JIL-2266 and H295R, treatment with BafA1 at both concentrations tested (10 and 20 nM) led to an increased expression of LC3-II and p62/SQSTM1, indicative of blockade on the autophagic flux, when compared to the respective vehicles and to cells treated with EBSS, a positive control for autophagy induction (**Figures 4A–C, E-G**).

Although the accumulation of LC3-II and p62/SQSTM1 was more pronounced at the higher BafA1 concentration (**Figures 4B, C, F–G**), this condition reduced cell density in both JIL-2266 and H295R cells after 48 h of treatment compared to the vehicle (**Figures 4D–H**).

Together, these results indicate that BafA1 concentration of 10 nM effectively decreases the autophagic flux having minimal effects on cell density in both ACC cell lines, validating its use in the downstream mechanistic studies.

#### 3.2.2 Impaired autophagy mitigates cell migration in JIL-2266 cells

**Figure 5:**
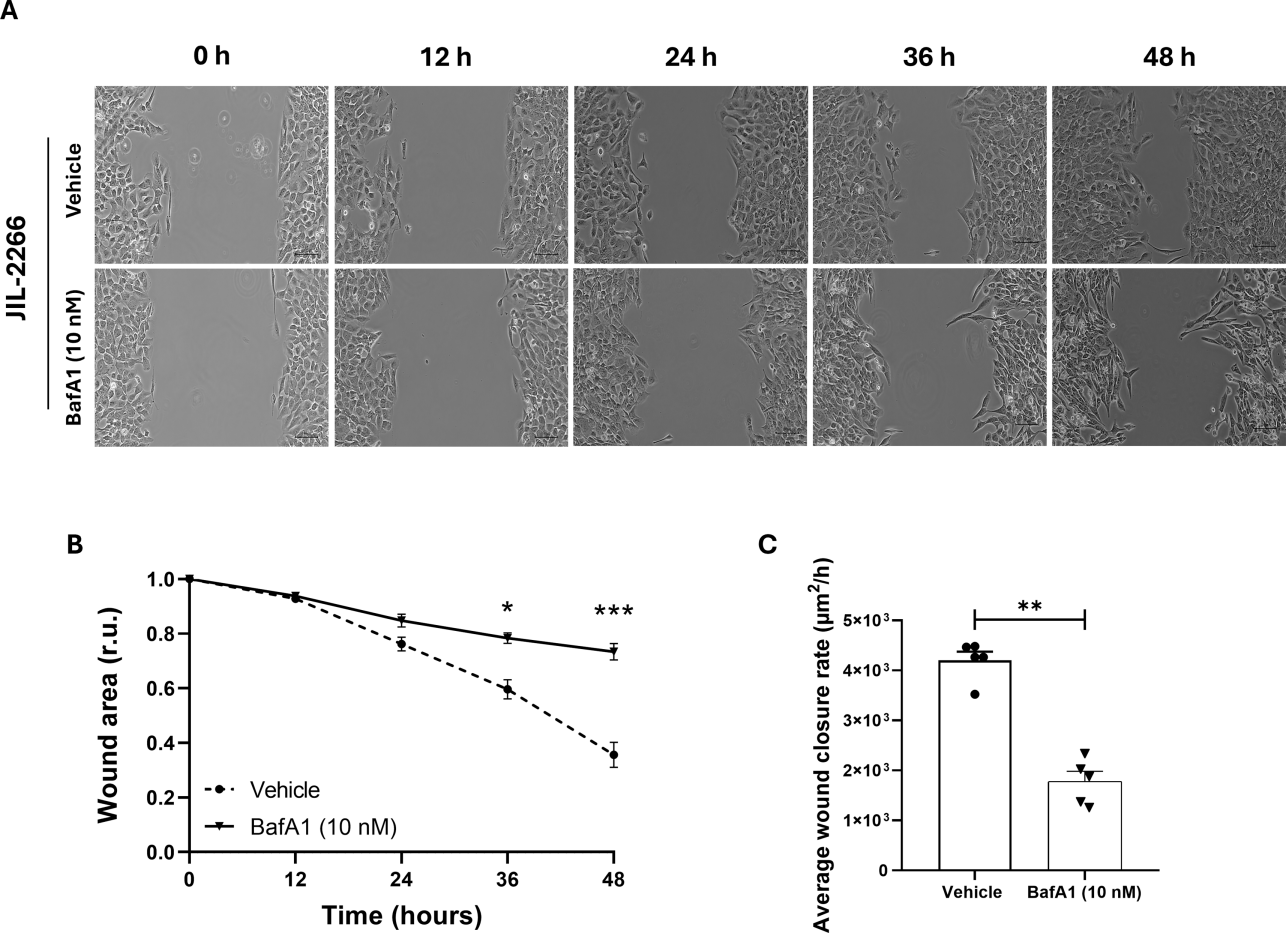
Analysis of ACC, adrenocortical carcinoma cells migration by *in vitro* wound healing assay. **(A)** Time-lapse microscopy images of wound closure of untreated (vehicle, upper panels) and treated with Bafilomycin A1 (BafA1, 10 nM; lower panels) JIL-2266 cells at 0, 12, 24, 36, 48 h after culture insert removal. **(B)** Quantification of the wounded area invaded during 48 h by untreated (vehicle) and treated (BafA1, 10 nM) JIL-2266 cells presented in r.u, relative units. Results represent the mean of 3 measures of each wounded area, obtained in 5 independent experiments (two-way ANOVA, followed by Sidak’s test: *p < 0.05, ***p < 0.0001). **(C)** Graph showing the average wound closure rate (µm^2^/h) in untreated (vehicle) and treated (BafA1, 10 nM) JIL-2266 cells (Mann-Whitney: **p < 0.01).

In JIL-2266 cells, clear migratory capacity was observed over time (**Figure 5A**). In this cell model, BafA1 treatment reduced wound healing compared with vehicle-treated controls. Specifically, the wound area was significantly higher in BafA1-treated cells when compared to the vehicle at 36 h and 48 h (**Figure 5B**), indicating impaired cell migration. Consistently, the average wound closure rate was significantly lower in BafA1-treated cells compared to the vehicle condition (1779.20 ± 202.81 *vs* 4201.20 ± 175.80 µm^2^/h, *p* = 0.007) (**Figure 5C**).

In contrast, migratory capacity was minimal in H295R cells with or without any treatment. Accordingly, no significant differences were detected in wound area over time or in the average wound closure rate between untreated (blank and vehicle) and BafA1-treated cells (**Supplementary Figure 3**).

#### 3.2.3 Impaired autophagy reduces the invasion capacity of ACC cell lines

**Figure 6:**
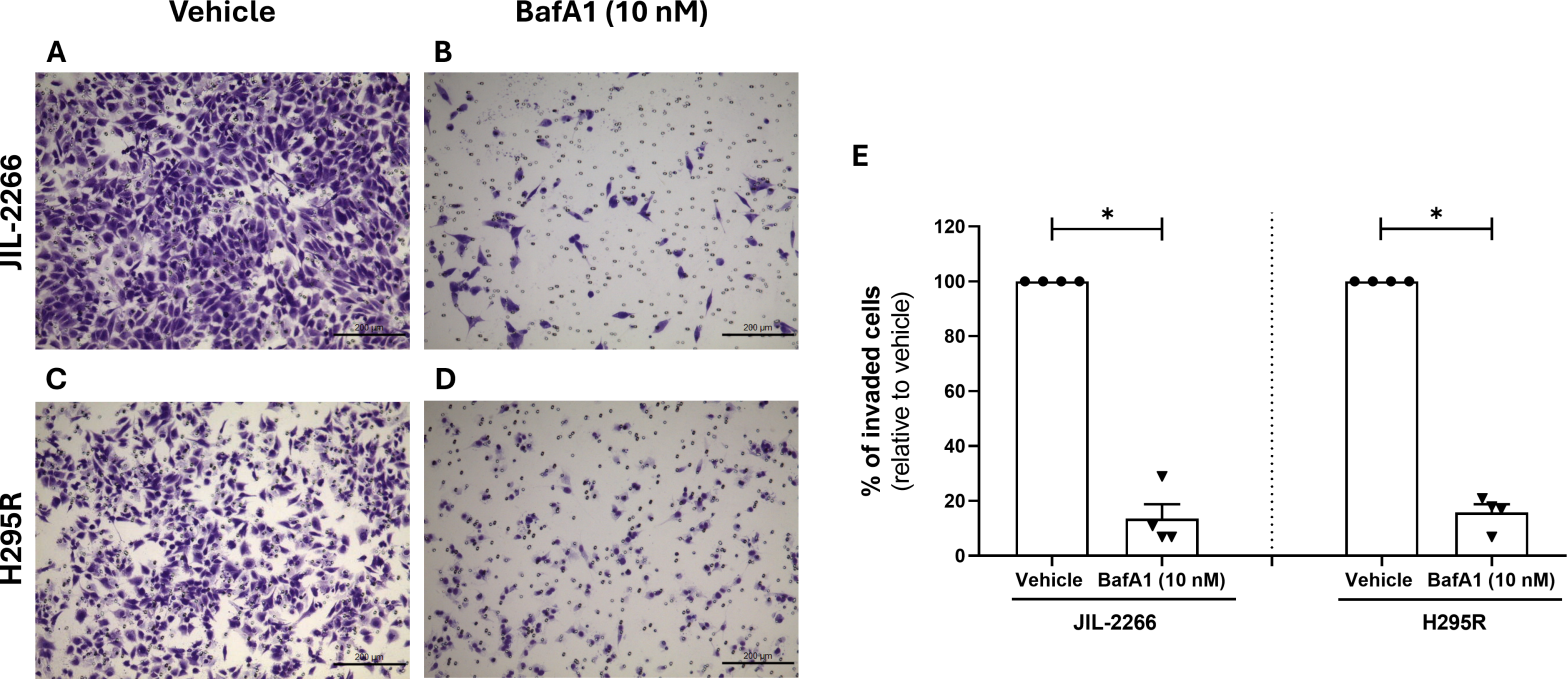
Effect of BafA1, Bafilomycin A1 on the invasive capacity of JIL-2266 and H295R cells assessed by *in vitro* Matrigel-Coated Transwell Assay. **(A–D)** Representative images of invaded cells stained with crystal violet are shown for JIL-2266 in **(A)** untreated (vehicle) (200×) and **(B)** treated (BafA1,10 nM) (200×) conditions; and for H295R cells in **(C)** untreated (vehicle) (200×) and **(D)** treated (BafA1,10 nM) (200×) conditions. **(E)** Percentage of invaded cells after 48 h, without and with treatment (BafA1, 10 nM), in JIL-2266 and in H295R cells. Comparison between percentage of invaded cells in vehicle and BafA1-treated cells in JIL-2266 (Mann–Whitney: *p < 0.05) and in H295R cell line (Mann–Whitney: *p < 0.05). Cells were counted from at least 5 random microscope fields for each condition in 4 independent experiments for each cell line.

Both cell lines demonstrated the ability to invade the matrigel matrix, which mimics *in vitro* basement membrane (**Figures 6A–D**). Interestingly, the treatment with BafA1 for 48 h significantly reduced the percentage of cell invasion when compared to the vehicle-treated condition in both JIL-2266 and H295R cell lines (**Figure 6E**).

## 4 Discussion

ACC is a rare but aggressive malignancy with heterogeneous clinical behavior and a poorly understood molecular pathophysiology (4, 30). In this study, we characterized the expression of key autophagy-related proteins in both ACA and ACC, aiming to clarify the potential contribution of autophagy to ACC pathophysiology.

In our study, LC3B expression, identified by a punctate staining pattern, was significantly higher in ACC compared to ACA. LC3B is one of the most widely used markers for monitoring autophagy (12). Although LC3 family comprises other isoforms, such as LC3A and LC3C, that may share similar functions to LC3B, these isoforms might also be involved in autophagy-unrelated processes such as signal transduction (31). Additionally, LC3 exists in two forms: LC3-I and lipidated LC3-II, the latter being associated with autophagosome maturation and acting as an adaptor protein by interacting with autophagic cargo via selective autophagy receptors, such as p62/SQSTM1 (32–34). Although IHC detection of LC3 cannot distinguish between LC3-I and LC3-II, a punctate staining is indicative of autophagosome formation, while diffuse cytosolic staining is associated with LC3-I form (12, 22, 35, 36). Accordingly, our analysis focused specifically on LC3B punctate staining pattern. Notably, this staining pattern was observed in the majority of ACC, whereas only 25% of ACA cases exhibited this feature. Furthermore, the percentage of cells displaying a punctate LC3B pattern was significantly higher in ACC compared to ACA, suggesting that LC3B punctate staining may be associated with malignancy.

Regarding p62/SQSTM1, we found that this protein was present in both the cytoplasm and nucleus of ACC and ACA cells. Cytoplasmic p62/SQSTM1 was observed in all ACC and ACA samples, with comparable levels in both groups. In ACC, higher cytoplasmic p62/SQSTM1 levels were observed in ACC cases classified as ENSAT stage III-IV. On the other hand, nuclear p62/SQSTM1 expression was significantly higher in ACA than in ACC. The relationship between p62/SQSTM1 expression and autophagy should be interpreted with caution, as this protein is also involved in several autophagy-independent functions. Beyond its role in autophagy, p62/SQSTM1 is also a central signaling hub, given its ability to interact with key proteins. Notably, p62/SQSTM1 has been shown to interact with transcriptional regulators, such as the transcription factor Nuclear Factor Erythroid 2-Related Factor 2 (NRF2), which results in the transcription of genes involved in oxidative stress defense. Additionally, p62/SQSTM1 can activate the Nuclear Factor Kappa B (NF-kB) pathway, promoting tumor cell proliferation and survival (37–39).

Although LC3B and p62/SQSTM1 nuclear were differentially expressed between ACC and ACA, their diagnostic performance was inferior, with lower sensitivity and specificity compared to those described for Ki-67 (40), a marker routinely used in the clinical practice for prognostic guidance in ACC (4).

To date, solely one study has evaluated LC3B and p62/SQSTM1 expression in ACC and ACA tissue samples. In that study, all ACC and most ACA cases (86%) showed negative LC3B immunostaining, while 54.8% of ACA and 41.2% of ACC were negative for p62/SQSTM1. This discrepancy in relation to our results may be attributed to methodological differences. While Kim et al. performed IHC in tissue microarrays (TMA), we analyzed whole tissue sections and subsequently selected tumor hotspots. We argue that this approach allows a more representative assessment of the expression status of both LC3B and p62/SQSTM1. Interestingly, the previously referred study reported the presence of isolated single positive cells (ISPC) with expression of LC3B and p62/SQSTM1.They found a significantly higher proportion of LC3B ISPC in ACC than in ACA and lower proportion of p62/SQSTM1 in ACA than in ACC, suggesting that higher LC3B expression appears to be associated with ACC, in line with our results (20). Consistent with these findings, malignancy associated LC3B expression has also been reported in prostate tumors (41).

Regarding ATG5, this is the first study to assess ATG5 expression by IHC in both ACC and ACA, and we found no association between ATG5 expression and ACC characteristics. Interestingly in our study, most ACC samples lacking ATG5 expression still exhibited positive LC3B staining. Our results can be explained by the fact that autophagy is a dynamic multi-step mechanism, and the static levels of ATG proteins evaluated by IHC may not accurately translate the autophagic flux in ACC. Indeed, the guidelines for the use and interpretation of assays for monitoring autophagy refers that IHC of ATG proteins in tissues to evaluate autophagy activity is an area of research which needs to be further explored to make specific recommendations and our study may also contribute to that (12).

Regarding the association between ATG5, LC3B and p62/SQSTM1 and ACC characteristics, we found no association between ATG5 expression and ACC characteristics. Consistent with our results, a pan-cancer analysis integrating *ATG5* gene expression and clinical information data from the Genotype-Tissue Expression Program (GTEx) and The Cancer Genome Atlas Program (TCGA) databases did not find a significant association between *ATG5* expression and overall survival in ACC patients (42). Nevertheless, in colorectal cancer high ATG5 expression was associated with worse overall survival (43), whereas in breast cancer, patients with high ATG5 levels exhibited better disease-free survival (44).

Regarding tumor functionality, we observed higher LC3B and nuclear p62/SQSTM1 expression in non-functioning ACC. A relationship between autophagy and steroidogenesis has been previously reported (45, 46). In a recent review, Bradic et al. highlighted the complex bidirectional interplay between autophagy and steroid hormone production. Steroids act as signaling molecules capable of regulating several molecular mechanisms, including autophagy, while autophagy, in turn, appears to modulate the availability of cholesterol, the precursor for steroid biosynthesis (47). Nevertheless, it is important to note that in non-functioning ACC, the absence of biochemical and clinically evident hormone excess, may reflect inefficient steroidogenesis, resulting in the secretion of steroids precursors rather than mature steroids (48, 49). Therefore, the classification of these tumors as non-functioning may not fully reflect their underlying steroidogenic activity. Consequently, no definitive conclusions can be drawn regarding the relationship between autophagy and steroidogenesis without the analysis of steroid precursors to more accurately assess steroidogenic activity.

In addition, higher LC3B expression in ACC was associated with early tumor stages and absence of distant metastasis. Consistent with LC3B findings, studies in pancreatic neuroendocrine neoplasms demonstrated that decreased *MAP1LC3B* transcripts was associated with distant metastasis (50, 51), despite reflecting gene rather than protein expression. Nevertheless, LC3B expression was evaluated by IHC in another study, and although no significant differences were observed, higher LC3B levels were reported in pancreatic neuroendocrine tumors without distant metastasis compared to those with metastasis (52). Similar association between LC3 expression and favorable clinical features, such as early tumor stages, have been described in esophageal cancer (53), with some studies reporting the association between higher LC3 levels with longer patient survival in hepatocellular carcinoma and colorectal cancer (54). However, contrasting findings have also been reported, demonstrating a correlation between higher LC3 expression and advanced tumor stages in gastric adenocarcinoma (55, 56), as well as poor prognosis in hepatocellular carcinoma, colorectal cancer and astrocytoma (57–59). In our study, although LC3B expression were associated with ENSAT stages, suggesting a potential prognostic role, Cox regression analysis did not demonstrate significant predictive value for survival in ACC patients.

It is important to note that increased LC3B expression in ACC without metastasis can reflect two scenarios, enhanced autophagic activity or impaired autophagic flux due to a blockage in the fusion of autophagosomes with lysosomes. A useful strategy to distinguish between these two scenarios involves *in vitro* modulation of autophagy using inhibitors of the fusion of autophagosome and lysosome, followed by the assessment of LC3-II levels by Western Blot in the presence and absence of these inhibitors (12, 60, 61). Therefore, the autophagic flux was inhibited in the two available ACC cell lines derived from primary human ACC, JIL-2266 and H295R, using BafA1, an autophagic inhibitor that blocks the fusion of autophagosomes with lysosomes (12). Aiming to understand the autophagy dynamics in ACC without metastasis, we further investigated whether alterations in autophagic flux could impact cell migration and invasion, which are key steps of the metastatic cascade (62, 63). Our *in vitro* findings indicate that autophagy inhibition significantly decreased migration in JIL-2266 cells. Although autophagy inhibition had no effect on H295R cells migration, this cell line exhibited limited migratory capacity under control conditions. This suggests that the lack of response is likely due to intrinsic characteristics of the H295R cell line, indicating that it may not represent an optimal model to study migration in ACC, as previously reported (64). Regarding the invasion assays, autophagy inhibition reduced cellular invasion in both cell lines. Taken together, these findings provide functional context for our immunohistochemical observations. The higher LC3B punctate staining observed in ACC tissues without metastasis may be consistent with impaired autophagic flux, potentially reflecting a disruption in autophagosome–lysosome fusion rather than increased autophagy induction. So, active autophagy seems to be associated with a more aggressive cellular phenotype in ACC, particularly by contributing to migration and invasion. Consistent with our results, thyroid cancer cells with active autophagy have shown that treatment with Lys05, a late-stage autophagy inhibitor, reduces cellular migration and invasion *in vitro* (65). Taken together, our *in vitro* results, along with the associations observed between LC3B expression and absence of metastasis in ACC and in pancreatic neuroendocrine tumors (50–52), as previously discussed, suggest that the relationship between LC3B and less aggressive disease may represent a broader feature of endocrine tumors rather than being specific to ACC. However, the limited number of studies specifically addressing autophagy in ACC warrants caution in interpreting these findings. Based on our *in vitro* results, modulation of this mechanism may potentially offer therapeutic benefit in ACC. Indeed, previous studies have explored the inhibition of autophagy as a potential treatment strategy in ACC. In CU-ACC2 cells, a cell line derived from a liver metastasis of an ACC patient, combined treatment with mitotane, the only pharmacological agent currently approved for ACC, and chloroquine, a late-stage autophagy inhibitor, resulted in increased PARP levels, indicating enhanced apoptosis compared with mitotane alone (66). Qin et al. demonstrated that concomitant treatment with cisplatin and chloroquine increased the apoptosis rate compared with cisplatin monotherapy *in vitro* and *in vivo* studies (21). Despite these encouraging preclinical findings, the clinical translation of autophagy-targeting strategies remains limited. Currently, chloroquine and hydroxychloroquine are the only Food and Drug Administration (FDA) approved drugs known to inhibit autophagy, and are indicated for the treatment of malaria as well as autoimmune diseases such as lupus erythematosus and rheumatoid arthritis (67, 68). Although they are not approved for cancer therapy, ongoing clinical trials are assessing their safety and efficacy as monotherapy and combination therapy, such as in pancreatic and breast cancer (69). However, these agents have not yet been specifically investigated in the clinical context of ACC. In ACC, additional preclinical studies are still needed to better define the contexts in which autophagy modulation may be therapeutically beneficial. In this regard, our findings provide a rationale for further investigation of autophagy inhibition as a potential therapeutic strategy in ACC, particularly given the observed reduction in cellular migration and invasion capacities following autophagy blockade *in vitro*. Nevertheless, further studies are still needed to elucidate the mechanistic link between autophagy and cell motility in ACC. Cell migration and invasion are highly dynamic processes that require coordinated cytoskeletal remodeling, focal adhesion turnover and extracellular matrix degradation (70). Given that autophagy has been implicated in protein turnover (71) and production of pro-invasive factors (72), one plausible hypothesis is that it may facilitates cell migration and invasion through the selective degradation of focal adhesion components, as well as through the autophagy-dependent production of pro-invasive factors. To test this hypothesis, a structured mechanistic framework could be implemented, combining genetic (e.g., ATG7 knockout) and pharmacological approaches to inhibit autophagy *in vitro*. Proteomic approaches may first provide insight into the regulation of adhesion-related proteins upon autophagy inhibition, followed by the assessment of focal adhesion dynamics by immunofluorescence staining of focal adhesion markers. Cytoskeletal organization could be further evaluated through phalloidin staining of actin to visualize its architecture. In addition, to directly assess the production of pro-invasive factors, both their intracellular expression and extracellular secretion should be quantified. Importantly, these *in vitro* findings should be complemented by *in vivo* validation using subcutaneous xenograft models derived from ACC ATG7-knockout cell lines. Such models would enable evaluation of tumor growth and metastatic potential in a more physiologically relevant setting. Furthermore, the analysis of organs commonly targeted by ACC metastasis (e.g., bone, lung, liver) would provide critical insight into the role of autophagy in metastatic dissemination (73).

The primary limitation of our study is the relatively small sample size, comprising 29 ACC and 20 ACA cases, which limited the statistical power for subgroup analyses. However, this reflects the rarity of ACC, which inherently restricts the availability of large and well-balanced cohorts. Nevertheless, the relatively small sample size and limited number of events (deaths) may have reduced the statistical power to predict associations between the expression of autophagy-related proteins and survival in ACC patients. In addition, the unequal distribution of ENSAT stages, with a predominance of advanced-stages ACC (ENSAT 3–4) due to challenges of early diagnosis of this disease, further limited the robustness of comparisons across disease stages and some clinicopathological features. These factors should be considered when interpreting our findings and may limit their generalizability, therefore, our results from subgroup analysis should be viewed as exploratory and require validation in larger and more balanced cohorts. Despite this limitation, our results strongly indicate that autophagy-related proteins, namely LC3B and p62/SQSTM1, play a role in ACC pathophysiology. Debnath et al. recently emphasized how critical it is to define the role of autophagy in different cancers and at different stages to understand how tumors rely on autophagy (17). In addition, our study extends beyond descriptive immunohistochemical observations by incorporating complementary *in vitro* experiments.

In conclusion, our findings indicate that autophagy may play a role in ACC molecular pathophysiology. A punctate LC3B expression accumulation, associated with less aggressive malignant features, namely the absence of metastasis, appears to result from a blockade at the late stages of autophagy. Whereas active autophagy may be associated with a more aggressive cellular phenotype in ACC, as suggested by its potential link to increased migration and invasion *in vitro*. Nevertheless, further studies are needed to confirm this association and clarify the underlying mechanisms.

## Data Availability

The raw data supporting the conclusions of this article will be made available by the authors, without undue reservation.
