# Supplementary material for: Tumor stage-dependent expression of autophagy proteins in adrenocortical carcinoma
Source: Front Endocrinol (Lausanne). 2026 May 18;17:1726834. doi: 10.3389/fendo.2026.1726834 (PMC13223127; doi:10.3389/fendo.2026.1726834)
Supplement: Supplementary file 1 [file Image1.pdf]

Supplementary Material

1 Supplementary Figure 1

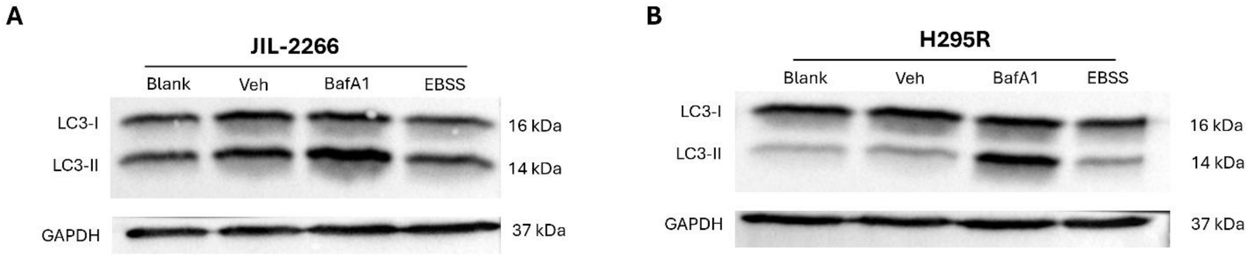

Supplementary Figure 1. Effect of Bafilomycin A1 (BafA1) on LC3-I and LC3-II expression in JIL-2266 and H295R cell lines. Cells were treated for 4 h with medium (blank), BafA1 (10 nM) or with DMSO (Vehicle), and with EBSS, an autophagy inducer. (A) Representative image of LC3-I, LC3-II and GAPDH Western Blot in (A) JIL-2266 and (B) H295R cells.
