# Supplementary material for: Tumor stage-dependent expression of autophagy proteins in adrenocortical carcinoma
Source: Front Endocrinol (Lausanne). 2026 May 18;17:1726834. doi: 10.3389/fendo.2026.1726834 (PMC13223127; doi:10.3389/fendo.2026.1726834)
Supplement: Supplementary file 2 [file Image2.pdf]

Supplementary Material

1 Supplementary Figure 2

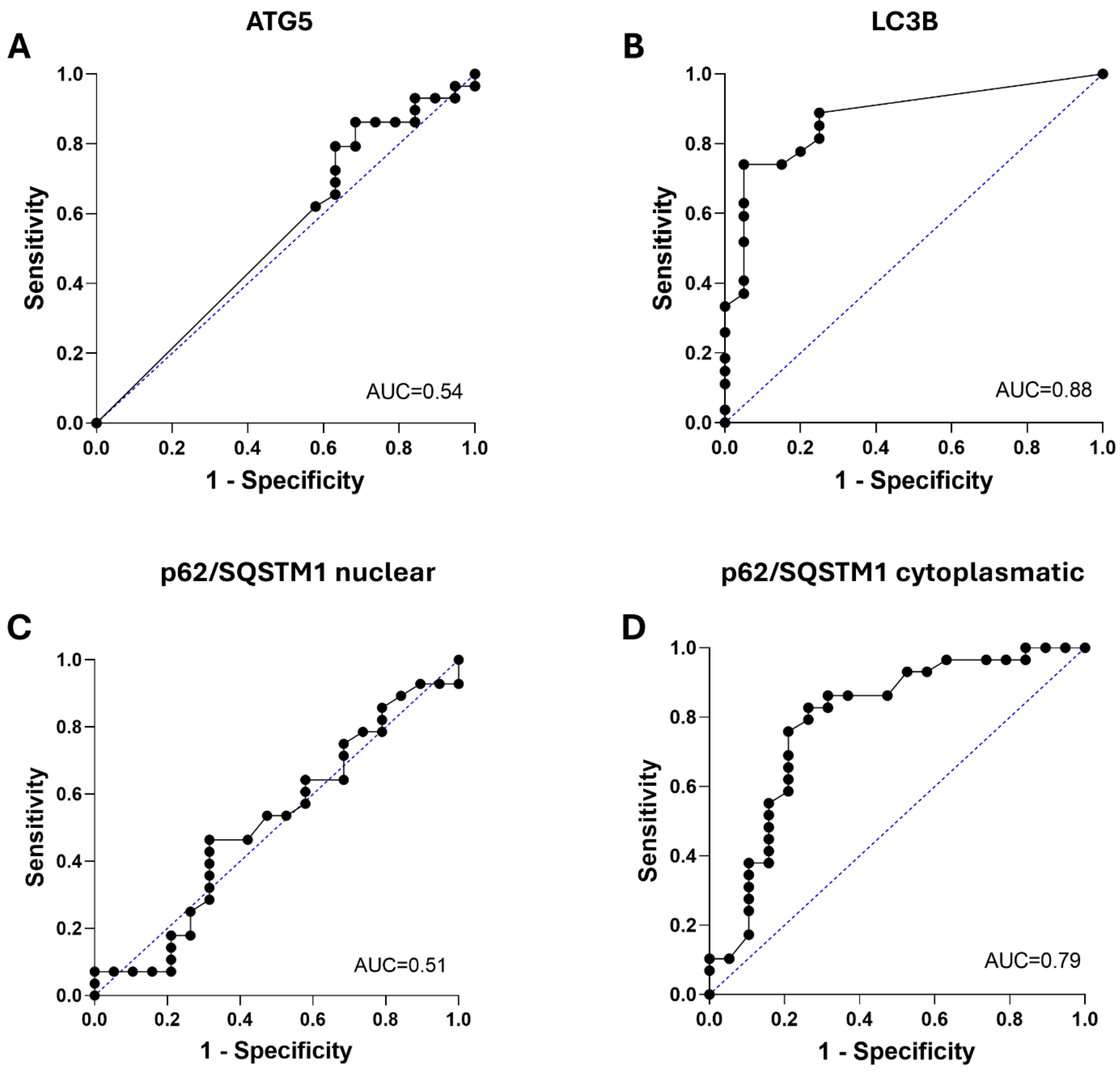

Supplementary Figure 2. Graphic representation of Receiving Operating Characteristic Curve (ROC) for (A) ATG5, (B) LC3B, (C) p62/SQSTM1 nuclear and (D) cytoplasmatic to distinguish between ACC and total ACA with the respective area under the curve (AUC).
