# Supplementary material for: Tumor stage-dependent expression of autophagy proteins in adrenocortical carcinoma
Source: Front Endocrinol (Lausanne). 2026 May 18;17:1726834. doi: 10.3389/fendo.2026.1726834 (PMC13223127; doi:10.3389/fendo.2026.1726834)
Supplement: Supplementary file 3 [file Image3.pdf]

Supplementary Material

1 Supplementary Figure 3

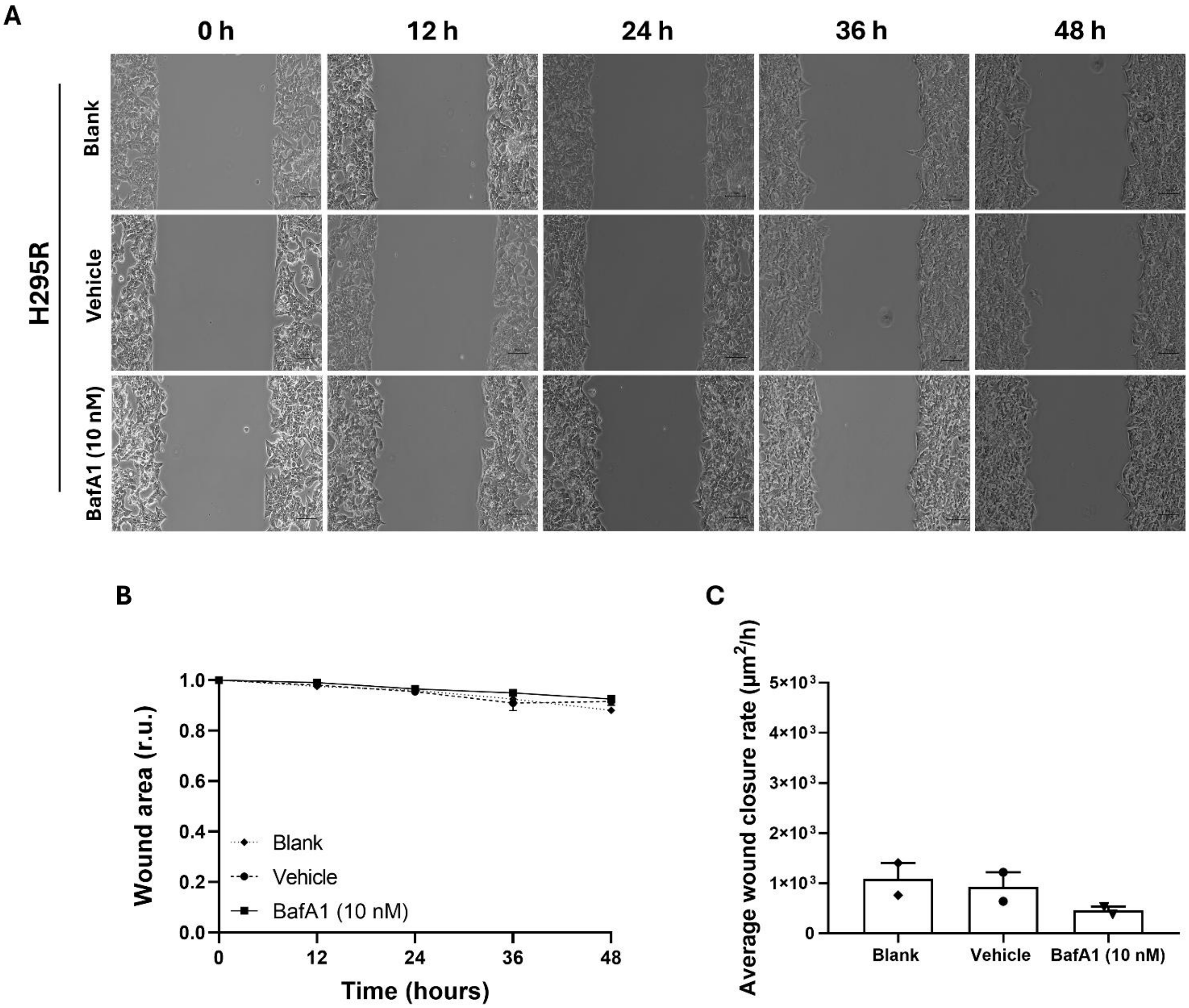

Supplementary Figure 3. Analysis of H295R cell migration by *in vitro* wound healing assay. (A) Time-lapse microscopy images of wound closure of untreated (blank, upper panels and vehicle, middle panels) and treated (Bafilomycin A1 (BafA1) 10 nM, lower panels) H295R cells at different time-points 0, 12, 24, 36, 48 h, after culture insert removal. (B) Quantification of the wounded area invaded during 48 h by untreated (vehicle) and treated (BafA1, 10 nM) in H295R cells presented in relative units (r.u). Results represent the mean of 3 measures of each wounded area, obtained in 2 independent experiments. (C) Graph showing the average wound closure rate ($\mu m^2/h$) in untreated and treated (BafA1, 10 nM) H295R cells.
