# Supplementary material for: Tumor stage-dependent expression of autophagy proteins in adrenocortical carcinoma
Source: Front Endocrinol (Lausanne). 2026 May 18;17:1726834. doi: 10.3389/fendo.2026.1726834 (PMC13223127; doi:10.3389/fendo.2026.1726834)
Supplement: Supplementary file 4 [file Table1.docx]

Supplementary Material

# Supplementary Table 1

Supplementary Table 1. Correlation analysis between autophagy related proteins in adrenocortical carcinoma.

| Autophagy-related proteins | | ATG5 | LC3B | nuclear  p62/SQSTM1 | cytoplasmatic  p62/SQSTM1 |
| --- | --- | --- | --- | --- | --- |
| ATG5 | | - | -0,06  (*p*=0.78) | 0.41  (*p*=0.03) | 0.07  (*p*=0.72) |
| LC3 | | -0,06  (*p*=0.78) | - | 0.14  (*p*=0.48) | -0.03  (*p*=0.87) |
| p62/SQSTM1 | nuclear | 0.41  (*p*=0.03) | 0.41  (*p*=0.03) | - | -0.34  (*p*=0.08) |
|  | cytoplasmatic | 0.07  (*p*=0.72) | -0.03  (*p*=0.87) | -0.34  (*p*=0.08) | - |

ATG5, autophagy-related protein 5; LC3B, microtubule – associated protein light chain 3 beta; p62/SQSTM1: Sequestosome 1; Correlation between ATG5 and p62/SQSTM1 nuclear: Spearmen test, *p*=0.03.
